# Internal Induction Heating for Local Heating in Injection Molding

**DOI:** 10.3390/polym17212906

**Published:** 2025-10-30

**Authors:** Thanh Trung Do, Huynh Duc Thuan, Tran Minh The Uyen, Nguyen Thanh Hon, Pham Son Minh, Tran Anh Son

**Affiliations:** 1 Faculty of Mechanical Engineering, Ho Chi Minh City University of Technology and Education, Ho Chi Minh City 71307, Vietnam; trungdt@hcmute.edu.vn (T.T.D.); uyentmt@hcmute.edu.vn (T.M.T.U.); nguyenthanhhon@caothang.edu.vn (N.T.H.); minhps@hcmute.edu.vn (P.S.M.); 2 Ho Chi Minh City University of Technology, Ho Chi Minh City 700000, Vietnam; tason@hcmut.edu.vn; 3 Vietnam National University—Ho Chi Minh City, Ho Chi Minh City 720325, Vietnam; 4 Military Technical Officer Academy (Tran Dai Nghia University), Ho Chi Minh City 727300, Vietnam

**Keywords:** injection molding, internal induction heating (In-IH), mold temperature control, thin-wall molding, polypropylene (PP), melt flow length

## Abstract

This study introduces Internal Induction Heating (In-IH) as an efficient method for local mold temperature control in thin-walled polypropylene (PP) injection molding. Unlike conventional systems that are slow and energy-intensive, the insert is integrated directly into the induction circuit in the In-IH system, generating eddy currents for rapid and localized heating. Numerical and experimental analyses were performed to examine the effects of insert geometry and heating parameters; it was found that thinner inserts achieved higher surface temperatures—the 0.5 mm insert reached ~550 °C, while the 2.0 mm insert reached only ~80 °C—confirming an inverse relationship between thickness and temperature. Narrower inserts (25 mm) concentrated heat more effectively, whereas wider ones yielded better temperature uniformity. The cooling conditions strongly affected the temperature gradients. Mold-filling experiments demonstrated that In-IH significantly improved the flowability of PP: at 180 °C, the 0.4 mm specimen achieved a flow length of 85.33 mm, compared with 43.66 mm for the 0.2 mm specimen. At 250–300 °C, all samples approached full filling (~100 mm). The simulation and experimental results agreed, with a maximum deviation of 10%, confirming that In-IH provides rapid, energy-efficient, and precise temperature control, thus enhancing melt flow and product quality for thin-walled PP components.

## 1. Introduction

Injection molding is a fundamental process in the polymer industry, enabling the mass production of complex components with high precision. However, challenges such as incomplete filling, weld lines, and warpage remain critical, especially in thin-walled products [1,2]. Among various mold temperature control methods, induction heating has emerged as a promising approach due to its rapid response, energy efficiency, and ability to enhance surface quality and mechanical strength [3,4,5,6].

Recent developments have explored multiple strategies for optimized mold temperature control. Hybrid systems combining induction with resistive or liquid-based cooling can achieve dynamic temperature cycling [7], while surface-heating techniques form the foundation of rapid temperature cycling (RTC) technologies [8,9]. Novel coil configurations, including high-frequency and ring-effect designs, enable improved heating uniformity and efficiency [10,11]. Compared with conventional resistive or fluid-based heating approaches, which often suffer from slow rates and high energy consumption [12,13,14], induction-based methods can achieve rapid temperature increases and effectively control weld-line formation [13,15,16].

Despite these advantages, achieving a uniform temperature distribution remains a major challenge due to factors such as coil–surface distance, magnetic field interference, and geometric effects [17,18,19]. Previous works have attempted to mitigate these issues by optimizing the coil design, adjusting the spacing, and employing multi-layer or flux concentrator systems [19,20,21,22]. Over the past five years, induction heating (IH) technology for injection molding has advanced significantly, with a particular focus on improving heating efficiency, achieving a uniform temperature distribution, and optimizing the coil and mold design. Recent studies, such as that of Li et al. (2023) [2], applied the Taguchi method and response surface methodology (RSM) to analyze the synergistic effects of induction heating parameters, establishing optimized operating conditions for enhanced energy efficiency. Similarly, Guo et al. (2023) [7] developed a rapid induction heating approach for PEEK microcellular injection molding, achieving a rapid surface temperature increase and precise control, making it suitable for fabrication of micro-structured parts.

Other research has emphasized the structural and electromagnetic optimization of molds. Muszyński et al. (2021) [3] and Poszwa et al. (2021) [5] investigated the use of induction-heated molds with movable sliders and selective induction heating to enhance part strength and surface quality. Additionally, Mrozek et al. (2020) [4] introduced the use of magnetic flux concentrators, which significantly increase the current density in targeted heating regions, thereby improving both heating speed and temperature uniformity.

Overall, studies published from 2020 to 2023 reflect a shift in research focus from merely achieving high temperatures within short durations to precisely controlling spatial temperature profiles and improving energy utilization. Current trends include the integration of IH with hybrid heating and cooling techniques (e.g., convection, liquid, or gas-assisted systems) to realize rapid temperature cycling (RTC). This integrated approach supports high-quality molding, shorter cycle times, and enhanced process stability in the manufacturing of advanced polymer and microstructured components.

To overcome the limitations of conventional external heating approaches, this study introduces Internal Induction Heating (In-IH) in which the insert is directly integrated into the induction circuit. This configuration enables localized, rapid, and precisely controllable heating, thereby improving the melt flow behavior and reducing the overall cycle time.

The primary objective of this study is twofold: first, to conduct a fundamental verification of the In-IH mechanism, and second, to demonstrate its effectiveness in enhancing the melt flow for thin-walled molded parts. The investigation focuses on how the insert geometry and heating parameters influence thermal behaviors, with the ultimate goal of establishing design guidelines for high-efficiency mold heating systems applicable to thin-walled injection molding.

## 2. Internal Induction Heating for Injection Mold

### 2.1. Internal Induction Heating Method

**Figure 1:**
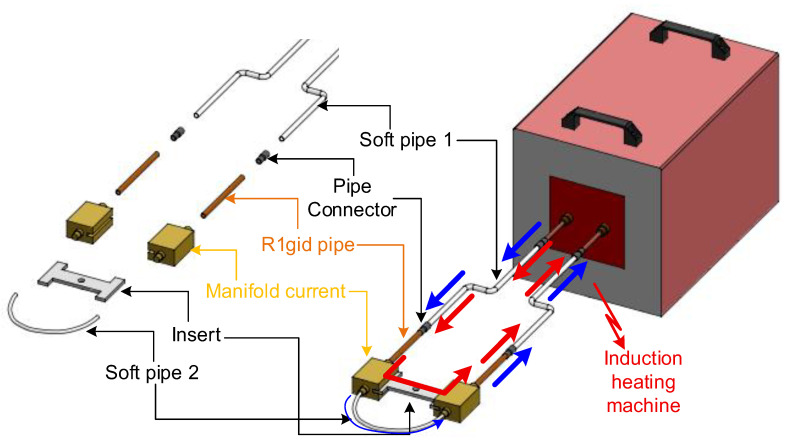
Principle of internal induction heating for mold temperature control.

The magnetic induction heating model for mold temperature control (Figure 1) consists of several key components. The induction heating machine supplies a high-frequency alternating current that drives the system. The first soft pipe comprises an outer plastic tube carrying cooling water and an inner copper wire that serves as a flexible electrical conductor; the second soft pipe contains only a plastic tube for water circulation dedicated to heat removal; and rigid pipes and pipe connectors ensure mechanical stability and secure integration of the system. The manifold current distributes and collects the electric current, guaranteeing uniform passage through the insert. The insert itself represents the mold component targeted for localized heating. During operation, cooling water flows through the piping system to regulate the temperature of surrounding parts, while the insert remains uncooled to allow for its efficient heating.

The operating principle relies on electromagnetic induction. When a high-frequency alternating current passes through the system, a time-varying magnetic field is generated and directly acts on the insert. This induces surface eddy currents within the insert, which are converted into heat via the Joule effect due to the intrinsic electrical resistance of the material. Consequently, the insert is rapidly and locally heated. The skin effect further concentrates the current density near the surface, ensuring that the highest temperatures develop precisely at the cavity wall where the polymer melt comes into contact.

After the induction heating stage, a cooling system is applied to rapidly lower the mold temperature and stabilize the process for the next injection cycle. The system typically consists of internal cooling channels or pipe circuits integrated within the mold body through which compressed air or cooling water is circulated. The cooling medium flows in the opposite direction of the heating current path, ensuring efficient heat extraction from the mold insert and surrounding steel structure.

Flexible soft pipes are used to connect the cooling channels to an external cooling unit or compressor, allowing for easy switching between heating and cooling stages. During the cooling phase, the airflow or water circulation absorbs residual heat from the mold surface, reducing the temperature uniformly and preventing thermal stress or deformation.

This cooling configuration ensures a cyclic thermal balance between the heating and solidification stages, shortens the overall cycle time, and maintains the dimensional accuracy and surface quality of the molded parts.

By combining localized induction heating at the insert surface with active water cooling elsewhere, the system achieves precise thermal control. The mold surface can be rapidly elevated to the desired temperature, thereby delaying premature solidification of the polymer melt and facilitating improved cavity filling. This approach not only enhances the surface quality but also reduces common defects such as weld lines and incomplete filling, making it particularly advantageous for thin-walled injection molding applications.

### 2.2. Simulation Method

#### 2.2.1. Simulation Model of Heating Process

**Figure 2:**
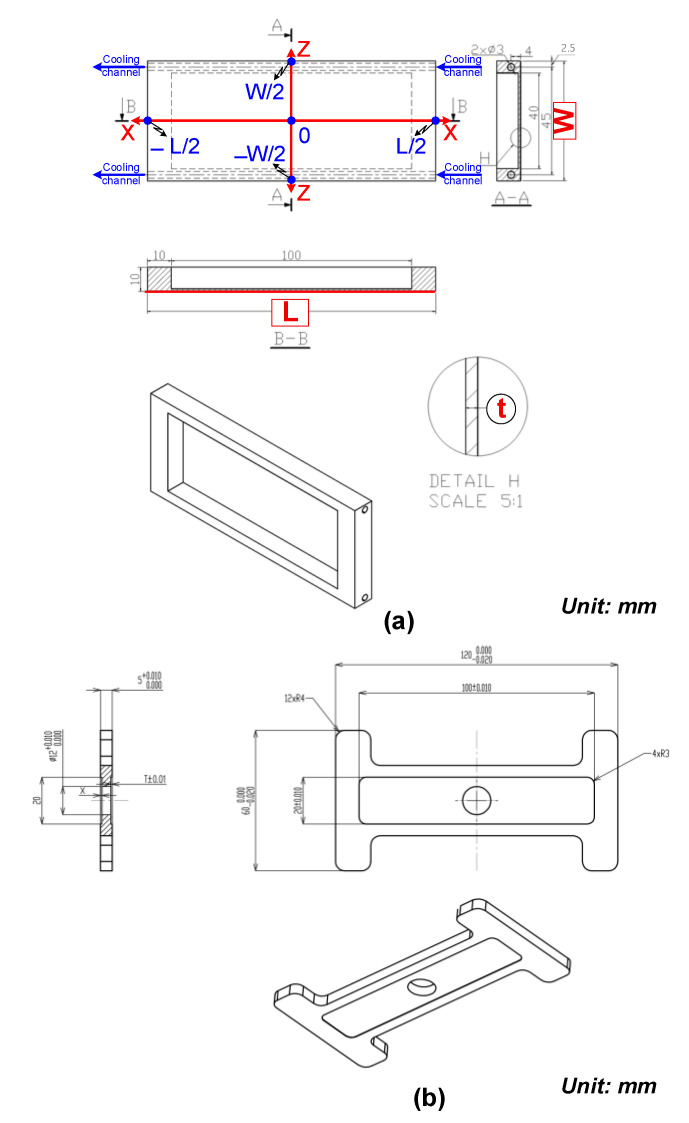
Insert dimensions of the evaluation model (**a**) and application model (**b**).

**Figure 3:**
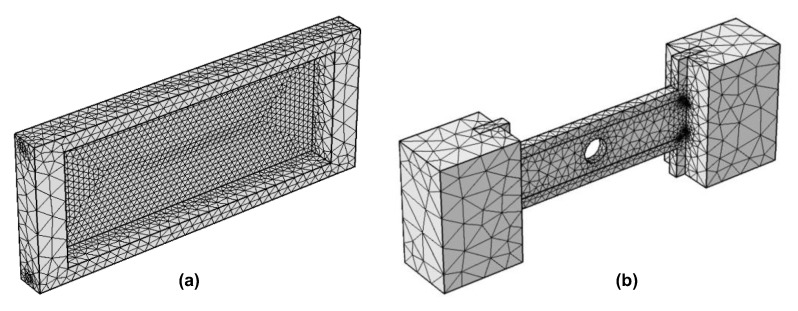
Mesh for cavity insert in the evaluation model (**a**) and application model (**b**).

The induction heating process for injection molds was simulated in detail using the COMSOL 5.0 Multiphysics software to analyze the temperature distribution and heating efficiency. To address this problem, two main physics modules were employed: Heat Transfer in Solids and Magnetic Fields. The simulation framework was designed to assess both the configuration and the operating parameters of the induction heating system. Two types of models were developed: an evaluation model, used to analyze the heating performance under simplified conditions, and a real cavity model, which represents the actual mold geometry. The insert dimensions corresponding to each model are presented in Figure 2. Furthermore, a mesh structure was applied to the cavity insert in both cases, as illustrated in Figure 3.

The simulations were governed by the fundamental physical equations of heat transfer and electromagnetism. Heat transfer within the solid domain was described by the energy balance equation, which accounts for conduction and thermal storage effects. The electromagnetic field was modeled using Maxwell’s equations, with an emphasis on the generation of eddy currents induced by the high-frequency alternating magnetic field. The coupling of these two physical models ensured that the conversion of electromagnetic energy into heat via the Joule effect was accurately captured.

This multiphysics approach provided a reliable tool to evaluate the effects of insert geometry, material properties, and boundary conditions on both the heating rate and the resulting temperature distribution, thereby enabling optimization of the induction heating approach for thin-walled injection molding applications:

(1) $$\rho C_{p}\frac{\partial T}{\partial t}+\rho C_{p}u.\nabla T+\nabla .(-k\nabla T)=Q$$

where ρ is the material density, *C_p_* is the specific heat capacity, *k* is the thermal conductivity, *u* is the velocity field, and *Q* is the volumetric heat source. In this study, *Q* corresponds to the Joule heating generated by the induced eddy currents within the insert.

To account for heat exchange with the environment or the cooling medium, a heat flux boundary condition is applied:

(2) $$q_{0}=h(T_{ext}-T)$$

where *q*_0_ is the heat flux across the boundary (W/m^2^); *h* is a convective heat transfer coefficient (W/m^2^·K) representing the intensity of heat exchange between the surface and the surrounding medium; *T_ext_* is the external temperature (i.e., the temperature of the surrounding environment or cooling medium in °C or K); and *T* is the surface temperature of the mold insert (°C or K).

A transient simulation was conducted using Air as the surrounding environment and steel as the assigned material for the insert. The detailed physical properties of all the materials are listed in Table 1, the overall model configuration and general simulation parameters are summarized in Table 2, and additional specifications for the real cavity model are provided in Table 3. The initial condition was defined at 30 °C and the analysis was performed over a time span of 0 to 9 s, with a minimum time step of 0.01 s to ensure sufficient temporal resolution.

#### 2.2.2. Simulation Model of Molding Process for Thin-Walled Products

**Figure 4:**
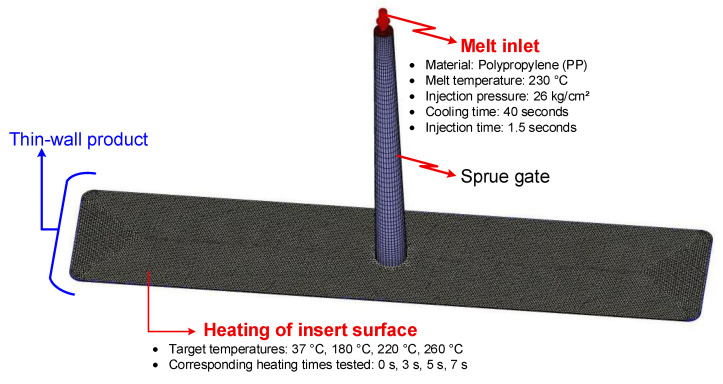
Mesh model for simulation of thin-wall injection molding assisted by internal induction heating.

The thin-walled injection molding process assisted by internal induction heating was simulated in detail to analyze the melt flow characteristics and optimize the key process parameters. The simulation framework was developed using Moldex3D, a specialized tool widely applied for polymer injection molding analysis. The geometries of the mold and product were discretized through meshing to convert the physical structures into numerical domains suitable for finite element analysis. The mesh model employed for thin-walled injection molding with internal induction heating is illustrated in Figure 4. Once the model geometry and simulation parameters were fully defined, the injection molding simulations were executed. Upon completion, the results were systematically examined to evaluate flow behaviors and assess the effects of process conditions on filling performance and part quality.

### 2.3. Experimental Method

#### 2.3.1. Heating Process via Internal Induction Heating with the Evaluation Model

To evaluate the influence of design parameters and operating conditions on the maximum temperature (Tmax), a series of systematic simulation experiments were conducted. Four key parameters were investigated: insert thickness (t), width (W), length (L), and coolant temperature. In each case, three parameters were held constant while the other was varied to analyze its individual effect. The specific values of the tested parameters are summarized in Table 4. This one-factor-at-a-time approach enabled a clear identification of the relative impact of each variable on the heating process, thereby supporting optimization of the In-IH system design for thin-walled injection molding applications.

**Figure 5:**
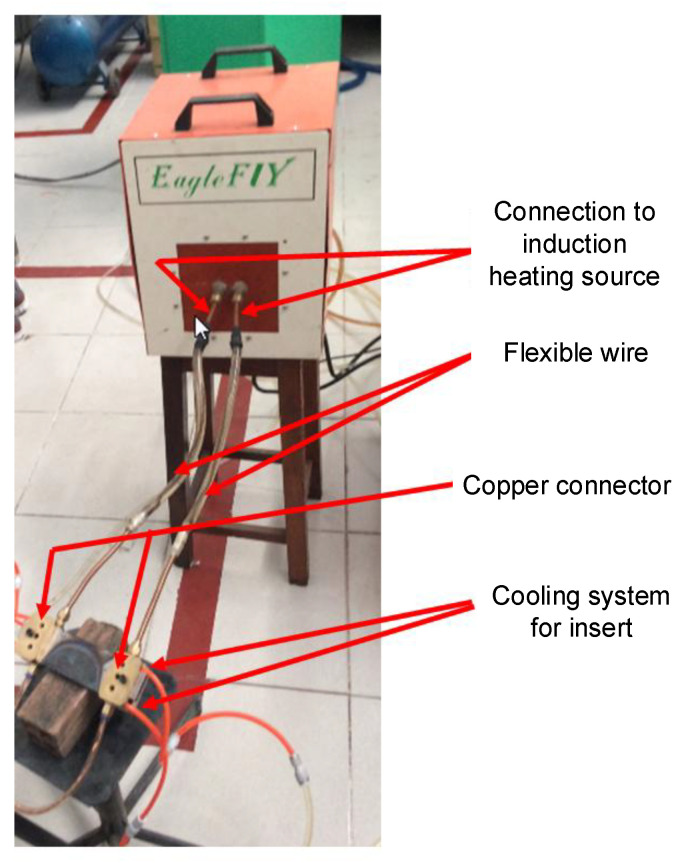
Model system for evaluation of the heating ability of the internal induction heating system.

**Figure 6:**
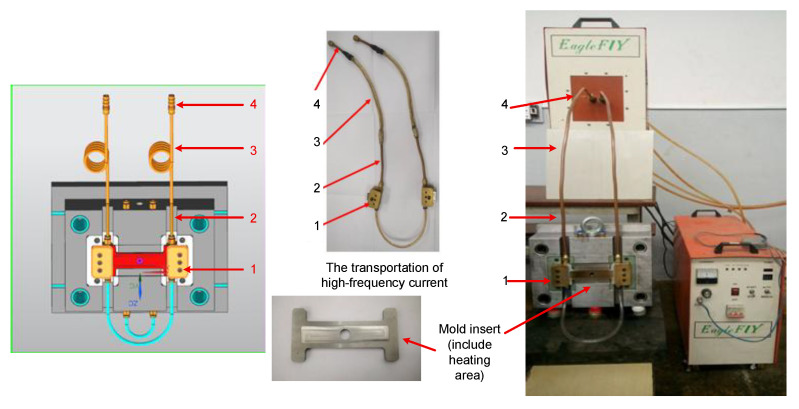
Model system for mold temperature observation consisting of an electrical contact (1), copper pipe (2), rubber pipe (3), and contact for connection with the induction heating source (4).

This study focused on investigating the internal induction heating (In-IH) process as an effective approach for controlling the mold surface temperature in injection molding. The heating model used to evaluate the system’s performance is illustrated in Figure 5, while Figure 6 provides a detailed view of the main components, including electrical contacts, copper tubing, rubber hoses, and the connectors to the induction heating power source. The system operates by supplying a high-frequency alternating current to the coil, which generates a strong magnetic field at the insert’s surface. To ensure efficiency and operational safety, water is continuously circulated to cool both the induction unit and the coil. The equipment is capable of delivering a maximum current of 150 A with a peak power output of 30 kW. The detailed technical specifications of the system are provided in Table 5.

To further improve accuracy, a Fluke TiS20 infrared camera (Fluke Corporation, Everett, Washington, DC, USA) was used in the experiment. The mold surface was coated with black paint and its emissivity was set to 0.95 to account for the low emissivity of steel. Temperature was recorded every 1 s during the induction heating period to monitor the thermal behavior.

#### 2.3.2. Molding Process

**Figure 7:**
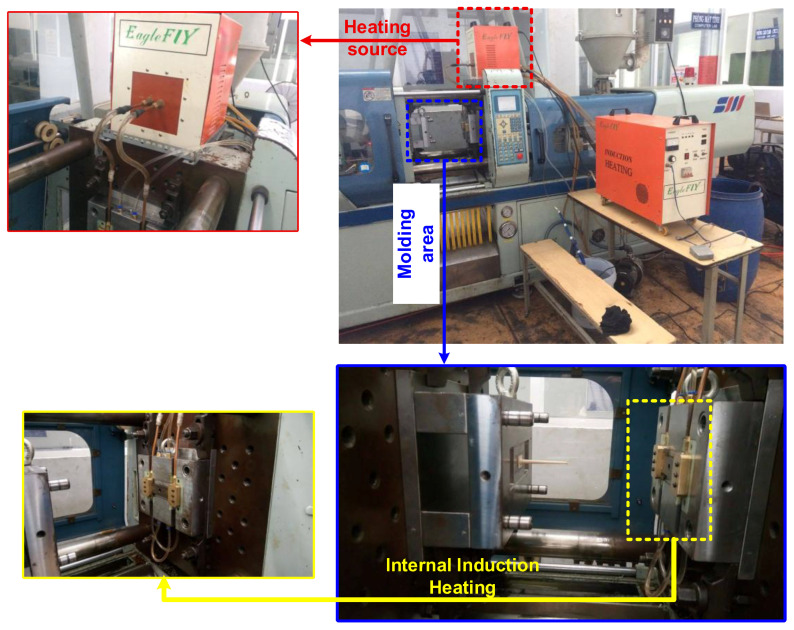
Injection mold system integrating internal induction heating.

Injection molding experiments were carried out using a mold system equipped with the proposed Internal Induction Heating (In-IH) system, as illustrated in Figure 7. The material employed was Advanced-PP 1100 N grade polypropylene (PP) homopolymer, supplied by Advanced Petrochemical Company (Al Jubail, Saudi Arabia). According to the manufacturer’s technical datasheet, the PP had a density of 0.90–0.91 g/cm^3^, specific heat of 1900–2300 J/(kg·K), melting temperature of 160–170 °C, molding shrinkage of 1.0–2.5%, melt flow index (MFI) of 2–30 g/10 min, and a coefficient of thermal expansion of (100–180) × 10^−6^ K^−1^. The typical injection molding parameters were as follows: barrel temperature of 180–240 °C, nozzle temperature of 180–230 °C, mold temperature of 20–60 °C, holding time of 3–12 s, holding pressure of 30–80 MPa, cooling time of 10–30 s, and screw speed of 30–80 rpm.

The molding process was operated under stable conditions to ensure consistency and reproducibility, with the key parameters set as follows: melt temperature of 230 °C, injection pressure of 26 kg/cm^2^, injection time of 1.5 s, and cooling time of 40 s.

Prior to injection, the insert surface was preheated with the In-IH system. Four target surface temperatures were examined: 37, 180, 220, and 260 °C, which correspond to heating durations of 0, 3, 5, and 7 s, respectively. The 0 s case represented the baseline condition where the mold remained at ambient temperature (37 °C).

The experimental plan was systematically structured. For each target surface temperature, ten consecutive injection molding trials were performed to ensure data reliability and minimize random error. In total, 150 molding experiments were conducted, covering the full range of operating conditions. This approach enabled a direct evaluation of the influence of mold surface temperature—precisely controlled through In-IH—on the filling performance of the thin-walled injection molding system. Furthermore, the repeated trials confirmed both the stability and the practical applicability of In-IH as an effective mold temperature control technique to improve product quality.

## 3. Results and Discussion

### 3.1. Local Heating Capability of the Insert Using the Evaluation Model

This section investigates the effects of geometric parameters and boundary conditions on the induction heating behaviors of mold inserts, as determined through numerical simulations, focusing on four key factors: insert thickness (t), width (W), length (L), and coolant temperature. By quantitatively analyzing these variables, this research aimed to provide deeper insights into the mechanisms of induction heating and to establish design and operational guidelines for future Internal Induction Heating (In-IH) systems.

#### 3.1.1. Effect of Insert Thickness (t)

Insert thickness has a profound influence on the temperature rise and heat concentration during internal induction heating. As the insert becomes thicker, both the induced current density and the maximum temperature decrease significantly due to changes in electromagnetic field penetration and thermal mass. In this study, four insert thicknesses (0.5, 1.0, 1.5, and 2.0 mm) were investigated, while the width (50 mm) and length (100 mm) were kept constant to isolate the influence of thickness.

**Figure 8:**
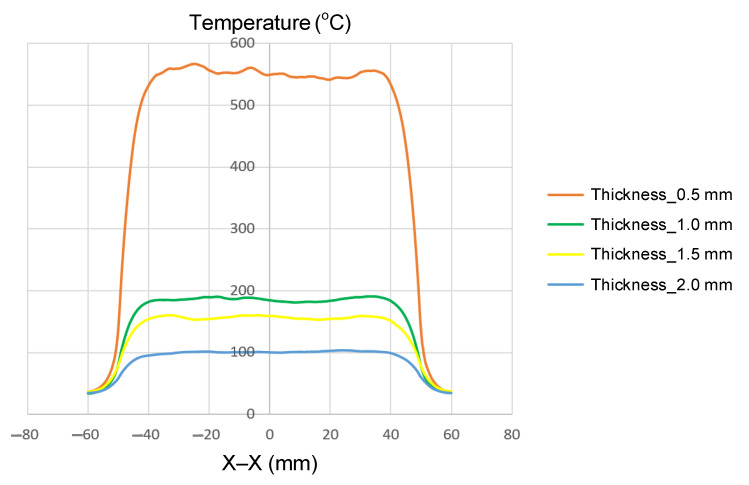
Summary of temperature on the X–X line for the case where the width (W) is 50 mm, the length (L) is 100 mm, and thickness (t) is 0.5, 1.0, 1.5, or 2.0 mm.

**Figure A1:**
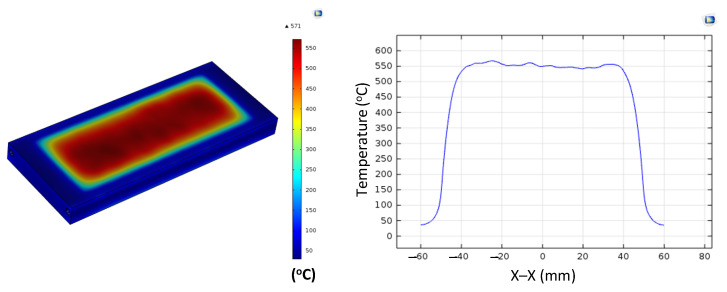
Simulation results and temperature distribution along the X–X line for the case where the thickness (t) is 0.5 mm, the width (W) is 5 mm, and the length (L) is 100 mm.

**Figure A2:**
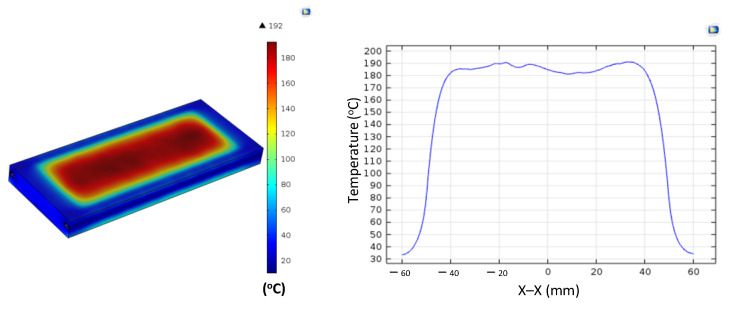
Simulation results and temperature distribution along the X–X line for the case where the thickness (t) is 1.0 mm, the width (W) is 50 mm, and the length (L) is 100 mm.

**Figure A3:**
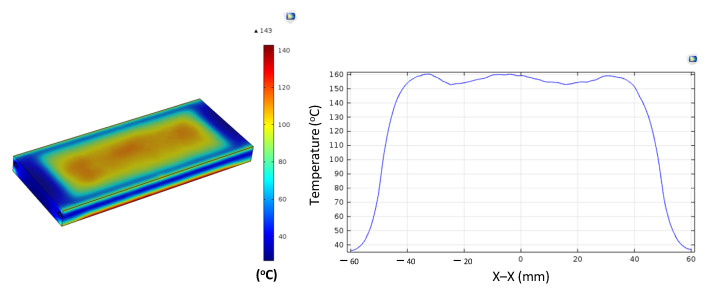
Simulation results and temperature distribution along the X–X line for the case where the thickness (t) is 1.5 mm, the width (W) is 50 mm, and the length (L) is 100 mm.

**Figure A4:**
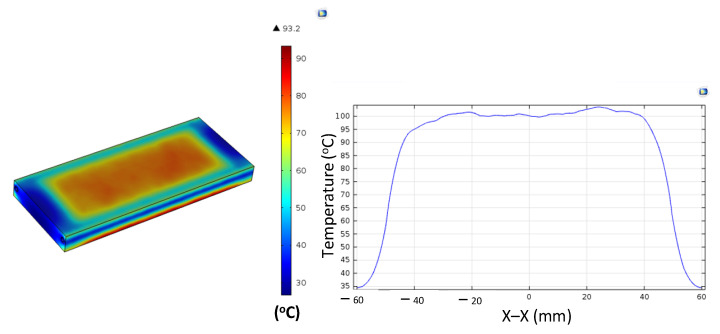
Simulation results and temperature distribution along the X–X line for the case where the thickness (t) is 2.0 mm, the width (W) is 50 mm, and the length (L) is 100 mm.

The simulation results (Figure 8 and Appendix A Figure A1, Figure A2, Figure A3 and Figure A4, and Table 6) reveal a clear inverse correlation between insert thickness and maximum temperature. When the insert was thinnest (t = 0.5 mm), the induced current was concentrated near the surface, resulting in rapid heating and a high peak temperature of approximately 550 °C. As the thickness increased to 1.0 and 1.5 mm, the maximum temperature decreased markedly to 200 and 160 °C, respectively, due to reduced current density and increased thermal diffusion. At the greatest thickness (2.0 mm), the temperature dropped further to around 100 °C, confirming that the heating efficiency declines sharply as the insert becomes thicker and the electromagnetic field penetrates less effectively.

The spatial temperature profiles also illustrate distinct differences in thermal uniformity. Thin inserts (0.5 mm) exhibited strong, localized heating at the surface with steep temperature gradients, while thicker inserts showed a more gradual temperature distribution but lower overall heating intensity. This behavior is consistent with the skin effect, where high-frequency induction currents are confined near the surface of conductive materials. Consequently, thinner inserts absorb and retain more surface heat, while thicker ones disperse energy into the bulk material, leading to slower and less efficient heating.

These findings align well with those of previous studies. Shokouhmand and Ghaffari [23] reported that increasing wall thickness in induction-heated cylinders intensified thermal fluctuations and reduced controllability. Similarly, other researchers have emphasized the need for modified coil designs or multi-frequency heating to ensure uniformity in thicker components [24].

In summary, the results confirm a strong inverse relationship between insert thickness and maximum temperature, providing quantitative evidence that supports fundamental induction heating theory. While thinner inserts significantly improve the heating efficiency, they also introduce challenges in achieving a uniform temperature distribution and maintaining structural integrity. Therefore, the optimal insert thickness should be selected jointly with an appropriate coil geometry and controlled heating parameters to balance the efficiency, temperature uniformity, and durability in industrial induction heating applications.

#### 3.1.2. Effect of Insert Width (W)

**Figure 9:**
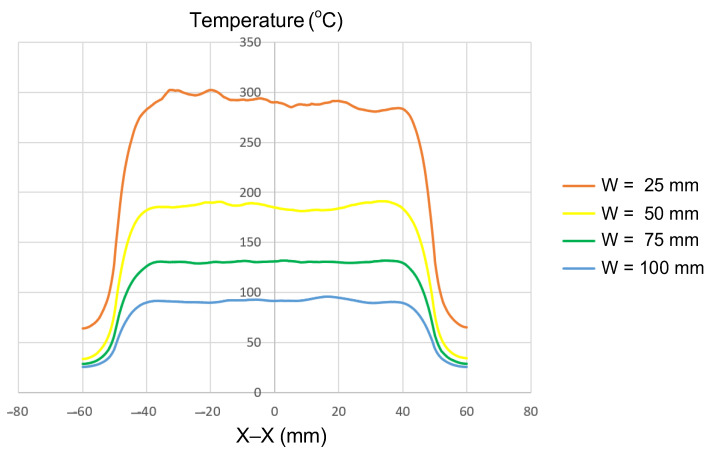
Summary of temperature on the X–X line for the case where the thickness (t) is 1.0 mm, the length (L) is 100 mm, and the width (W) is 25, 50, 75, or 100 mm.

**Figure A5:**
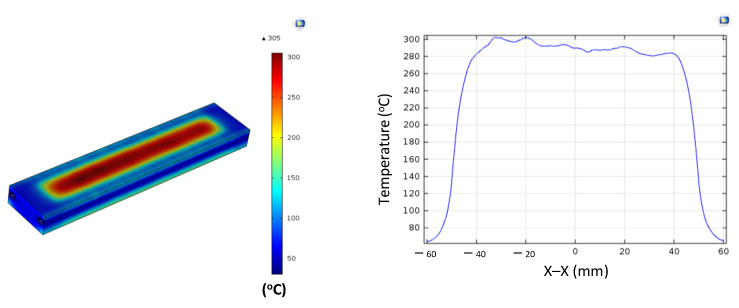
Simulation results and temperature distribution along the X–X line for the case where the thickness (t) is 1.0 mm, the width (W) is 25 mm, and the length (L) is 100 mm.

**Figure A6:**
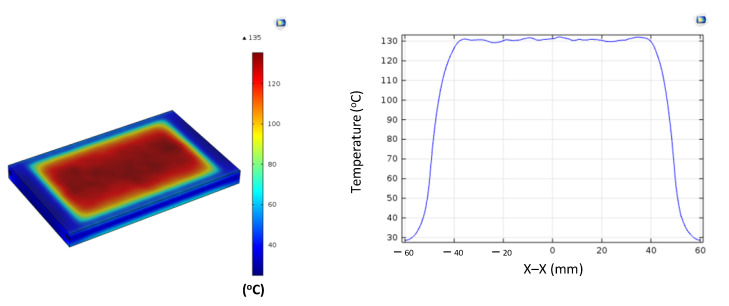
Simulation results and temperature distribution along the X–X line for the case where the thickness (t) is 1.0 mm, the width (W) is 75 mm, and the length (L) is 100 mm.

**Figure A7:**
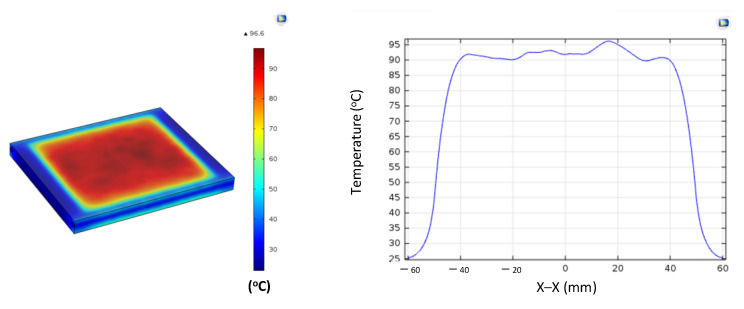
Simulation results and temperature distribution along the X–X line for the case where the thickness (t) is 1.0 mm, the width (W) is 100 mm, and the length (L) is 100 mm.

Insert width is one of the most influential geometric parameters governing how induction energy is distributed across the mold surface. In this study, simulations were conducted with a fixed insert thickness of 1.0 mm and a length of 100 mm, while the width was varied from 25 to 100 mm (Figure 2a). The results (Figure 9 and Appendix A Figure A5, Figure A2, Figure A6 and Figure A7, and Table 7) showed a clear dependence of the maximum temperature on insert width. For the narrowest insert (W = 25 mm), the maximum temperature exceeded 300 °C. As the width increased to 50, 75, and 100 mm, the maximum temperature decreased significantly, reaching approximately 190, 140, and 100 °C, respectively. This trend reveals a deeper physical mechanism behind the induction heating process. When the insert width is small, the electromagnetic field becomes more concentrated near the surface, leading to a higher induced current density, and thus, a greater localized heat generation rate. The smaller surface area acts as a confined region for electromagnetic energy, resulting in a rapid increase in temperature and a steeper thermal gradient. Conversely, as the insert width increases, the magnetic flux spreads over a broader area, causing the induced current to distribute more evenly and reducing its density. This dispersion lowers the power density per unit area, thereby decreasing the peak surface temperature and overall heating efficiency.

Furthermore, wider inserts experience enhanced heat dissipation toward the surrounding material, which intensifies thermal losses and limits the attainable temperature. The combination of electromagnetic field dispersion and conductive heat loss explains the nonlinear reduction in maximum temperature with increasing width. Therefore, optimizing the insert width is essential to balance energy concentration, temperature uniformity, and thermal stability during induction heating for molding applications.

These results are consistent with previous reports. For example, COMSOL-based studies have demonstrated that narrow coils (0.02–0.1 m) concentrate current strongly at the edges due to the skin effect, producing localized hot spots and higher surface temperatures [25]. Another study confirmed that under equal input power, narrower coils transferred energy faster and more effectively, while wider coils reduced heating rates [26].

However, a narrow insert is not always the optimal choice. The analysis indicated that wider inserts (W ≥ 75 mm) provide a more uniform temperature distribution, despite lower maximum values. This uniformity is advantageous in applications where stable surface heating is required. Similar findings have been reported in studies on the induction welding of composite plates, where widths between 6.25 and 7.5 cm yielded the most uniform heating. In contrast, very narrow (≈5 cm) or very wide (≥8.75 cm) inserts produced less favorable uniformity, with excessively wide samples tending to concentrate heat at the center while reducing efficiency at the edges [27].

These observations suggest that greater width does not inherently guarantee improved uniformity. Instead, there exists an optimal width range where maximum temperature and uniform distribution are balanced. The present study therefore adds quantitative evidence to established principles: insert and coil geometry directly affect the heating rate, peak temperature, and thermal uniformity.

In summary, controlling the insert width is critical not only for heating efficiency but also for temperature distribution quality. Narrow inserts are suitable for rapid, localized heating, whereas wider inserts favor stable and uniform thermal fields. Thus, selecting an optimal insert width is essential to simultaneously satisfy performance, speed, and quality objectives in industrial induction heating applications [25,26,27].

**Figure 10:**
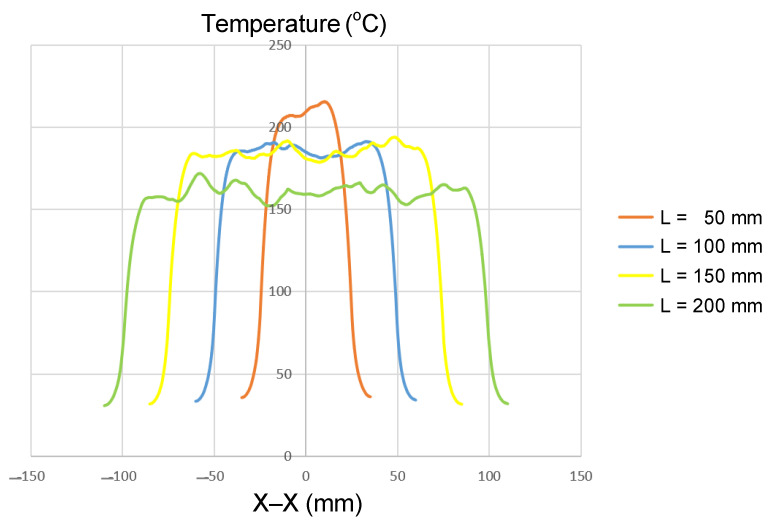
Summary of temperature on the X–X line for the case where the thickness (t) is 1.0 mm, the width (W) is 50 mm, and the length (L) is 50, 100, 150, or 200 mm.

**Figure A8:**
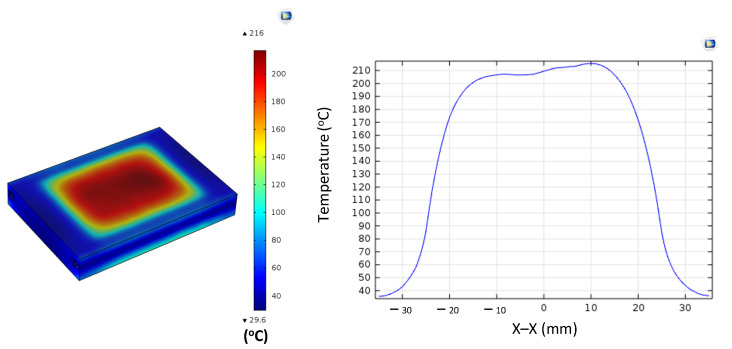
Simulation results (a) and temperature distribution along the X–X line (b) for the case where the thickness (t) is 1.0 mm, the width (W) is 50 mm, and the length (L) is 50 mm.

**Figure A9:**
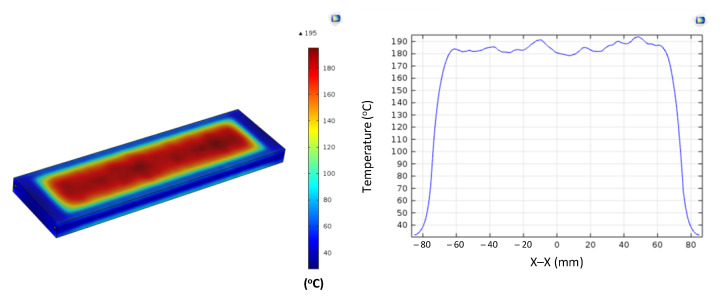
Simulation results and temperature distribution along the X–X line for the case where the thickness (t) is 1.0 mm, the width (W) is 50 mm, and the length (L) is 150 mm.

**Figure A10:**
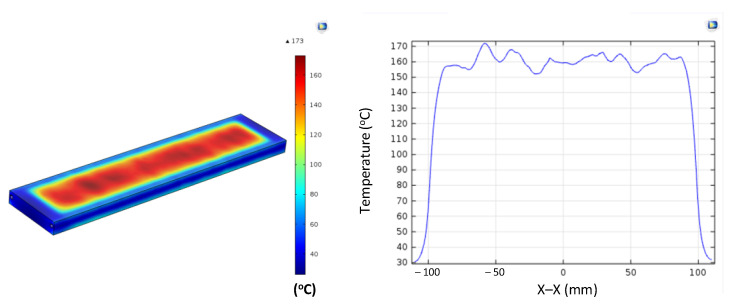
Simulation results and temperature distribution along the X–X line for the case where the thickness (t) is 1.0 mm, the width (W) is 50 mm, and the length (L) is 200 mm.

Insert length plays a critical role in determining how induction power is distributed and how heat conduction develops along the axial direction. In this study, four insert lengths (50, 100, 150, and 200 mm) were investigated, while the other geometric parameters were kept constant, namely, t = 1.0 mm and W = 50 mm (Figure 2a). The simulation results (Figure 10 and Appendix A Figure A8, Figure A2, Figure A9 and Figure A10, and Table 8) revealed that insert length strongly influences both the maximum temperature and thermal uniformity during induction heating.

For the shortest insert (L = 50 mm), the maximum temperature exceeded 210 °C, but this was accompanied by a steep temperature gradient, particularly near the edges. Increasing the length to 100 and 150 mm slightly reduced the maximum temperature to approximately 190–200 °C, while simultaneously expanding the stable heating zone. For the longest insert (L = 200 mm), the maximum temperature dropped further to about 160 °C; however, the temperature distribution along the length became the most uniform. Overall, extending the length from 50 mm to 200 mm reduced the maximum temperature by about 20–25%, while significantly improving uniformity.

This trend can be explained by the balance between energy density and axial heat conduction. Short inserts concentrate all input power within a small volume, producing a high energy density and elevated peak temperatures. In contrast, longer inserts spread the same input power across a larger volume, reducing the peak value but allowing for axial conduction to smooth out the temperature profile. Additionally, the relative contribution of edge losses decreases with length, further supporting thermal uniformity.

These findings are consistent with those of earlier studies. Tavakoli et al. [28] observed that when the workpiece height is smaller than or equal to the coil height, overheating at the ends becomes pronounced, leading to non-uniform heating. Although focused on height, the same principle applies to length: small inserts confined within the heating zone show localized thermal concentration, whereas longer inserts distribute heat more evenly. Similarly, Martín et al. [27] demonstrated that increasing specimen width reduced edge effects and improved uniformity in the induction welding of composite plates—a phenomenon analogous to the present observations regarding insert length.

In summary, the results highlight a clear trade-off: short inserts are suitable for applications requiring high peak temperatures localized in small areas, such as enhancing local flow in thin-wall injection molding; on the other hand, longer inserts are advantageous for achieving stable and uniform heating across larger surfaces, as required in surface treatment or induction welding, despite their lower peak temperature. Consequently, insert length—or, equivalently, the effective heating length of the coil—must be carefully optimized in system design. Such optimization enables a balance between localized heating and thermal uniformity, ultimately enhancing the industrial applicability of induction heating technology [27,28].

#### 3.1.3. Effect of Coolant Temperature

**Figure 11:**
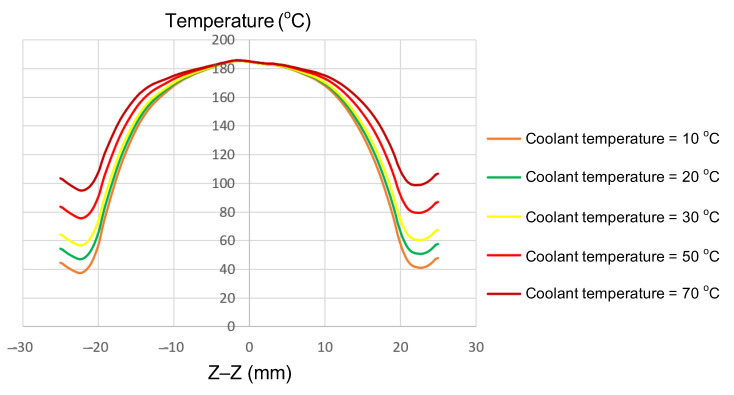
Summary of temperature on the Z–Z line for the case where the thickness (t) is 1.0 mm, the width (W) is 50 mm, the length (L) is 100 mm, and the coolant temperature is 10, 20, 30, 50, or 70 °C.

**Figure A11:**
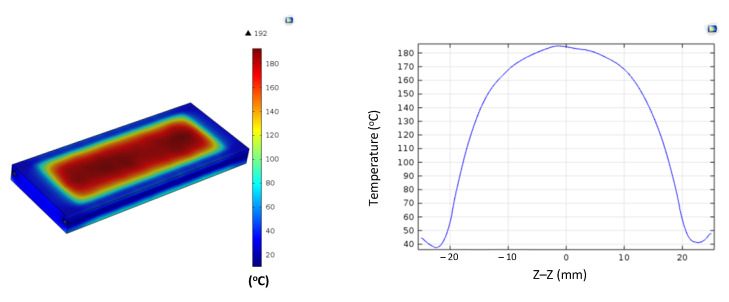
Simulation results and temperature distribution along the Z–Z line for the case where the thickness (t) is 1.0 mm, the width (W) is 50 mm, the length (L) is 100 mm, and the coolant temperature is 10 °C.

**Figure A12:**
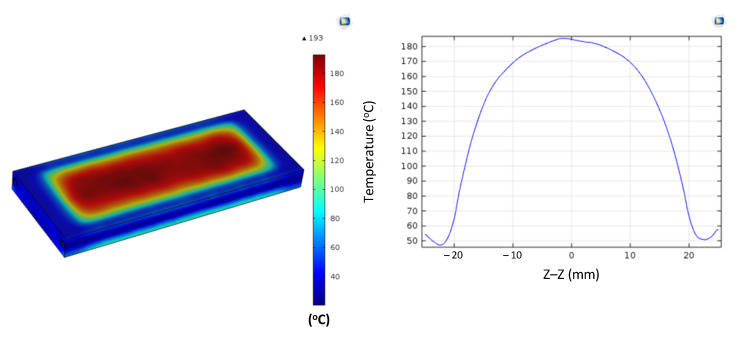
Simulation results and temperature distribution along the Z–Z line for the case where the thickness (t) is 1.0 mm, the width (W) is 50 mm, the length (L) is 100 mm, and the coolant temperature is 20 °C.

**Figure A13:**
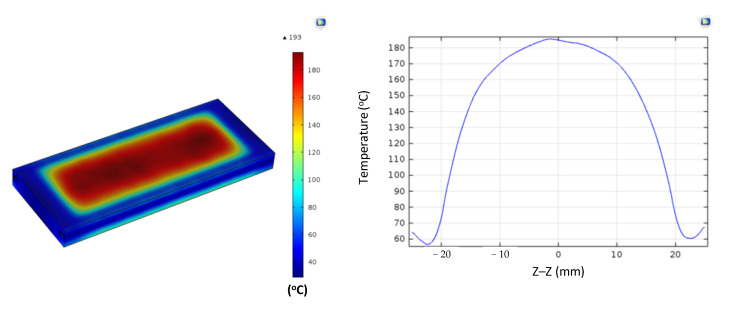
Simulation results and temperature distribution along the Z–Z line for the case where the thickness (t) is 1.0 mm, the width (W) is 50 mm, the length (L) is 100 mm, and the coolant temperature is 30 °C.

**Figure A14:**
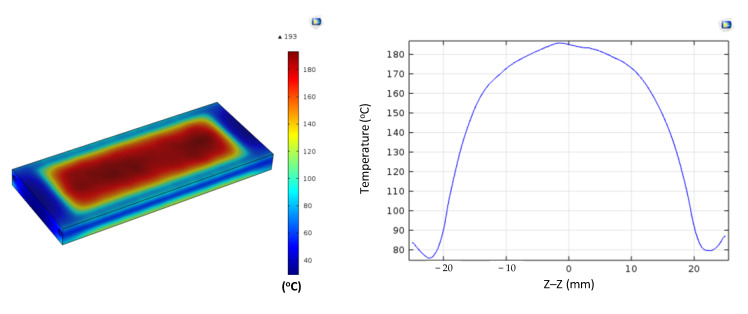
Simulation results and temperature distribution along the Z–Z line for the case where the thickness (t) is 1.0 mm, the width (W) is 50 mm, the length (L) is 100 mm, and the coolant temperature is 50 °C.

**Figure A15:**
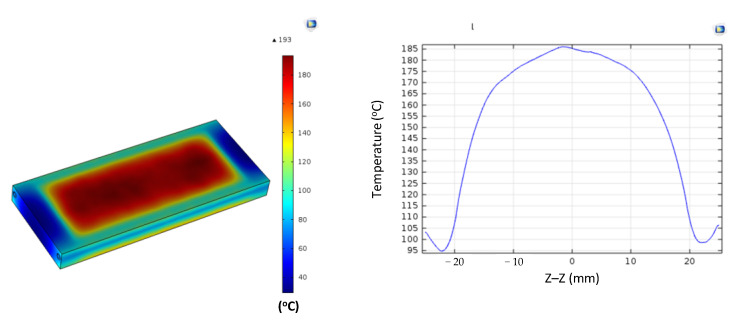
Simulation results and temperature distribution along the Z–Z line for the case where the thickness (t) is 1.0 mm, the width (W) is 50 mm, the length (L) is 100 mm, and the coolant temperature is 70 °C.

The coolant temperature functions as a critical boundary condition, directly influencing the rate of heat dissipation and the temperature gradient between the central and edge regions of the insert. In this study, the coolant temperature was varied from 10 to 70 °C to evaluate its effects on thermal uniformity and gradient. Simulations were performed on inserts with fixed dimensions (t = 1.0 mm, W = 50 mm, L = 100 mm; Figure 2a). The parameter of interest was the coolant temperature, which was examined at 10, 20, 30, 50, and 70 °C. The results (Figure 11 and Appendix A Figure A11, Figure A12, Figure A13, Figure A14 and Figure A15, and Table 9) revealed distinct thermal behaviors across these conditions. At the insert center, the maximum temperature remained nearly constant, at 180–185 °C, showing little sensitivity to coolant variation. In contrast, edge temperatures increased significantly, rising from 115–120 °C at a 10 °C coolant temperature to 165–170 °C at 70 °C. Consequently, the center-to-edge temperature difference narrowed from 60–70 °C to just 15–20 °C, demonstrating that boundary condition control is an effective approach to enhance thermal uniformity.

This phenomenon can be explained by differences in heat transfer mechanisms between the center and edges. At the center, induction power is highly concentrated and quickly reaches saturation, rendering it relatively insensitive to cooling effects. However, the edges are strongly affected by conduction, convection, and radiation, making them more responsive to coolant changes. With elevated coolant temperatures, heat loss at the edges decreases while lateral thermal diffusion is enhanced, thereby reducing the overall gradient.

These trends are consistent with those reported in previous studies. Chen et al. [7] confirmed that boundary conditions strongly influence thermal gradients and, ultimately, product quality. Shokouhmand and Ghaffari [23] highlighted the complex interaction between induction heating and spray cooling, emphasizing the decisive role of the cooling environment. Guerrier and Hattel [29] reported that cooling rates decline as a steady state is reached, reinforcing the importance of boundary conditions. Lin et al. [11] observed temperature differences of approximately 15–20 °C across heated plates after 10 s, closely matching the present findings. Similarly, Nian et al. [1] noted non-uniform distributions caused by the proximity effect and proposed solutions to improve uniformity, indirectly supporting the effectiveness of coolant temperature adjustments.

In summary, increasing the coolant temperature from 10 to 70 °C reduced the center–edge temperature difference from 60–70 to 15–20 °C, providing clear quantitative evidence of the effectiveness of boundary condition adjustment in improving thermal uniformity. From an application perspective, these findings establish a foundation for designing induction heating systems that are not only energy-efficient but also capable of delivering a uniform thermal distribution—an essential factor for improving the quality of thin-walled injection-molded products [1,7,11,23,29].

#### 3.1.4. Experimental Verification

**Figure 12:**
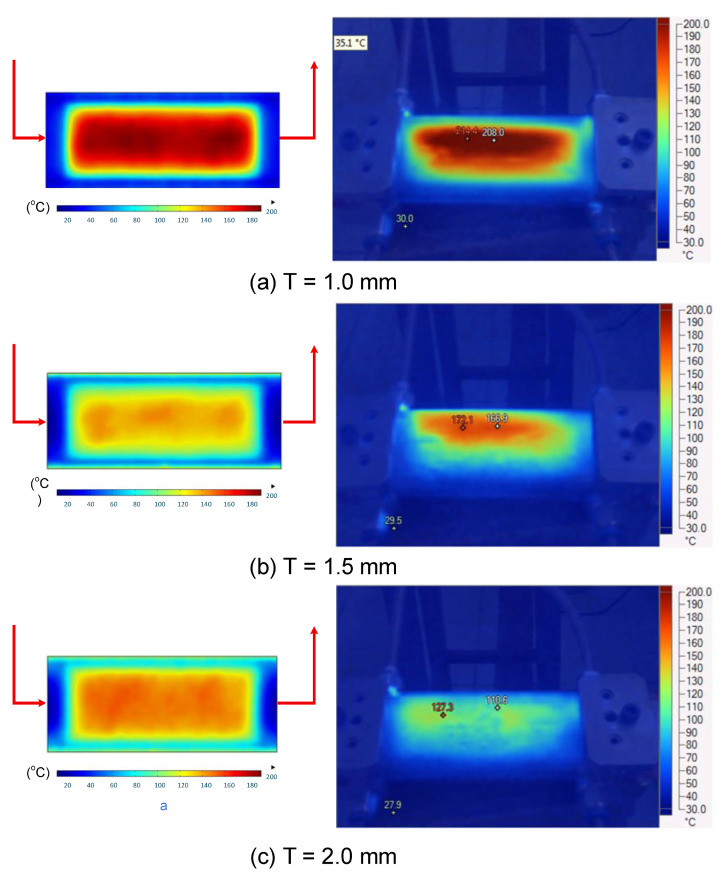
Simulation and experimental measurement results for the case where thickness (t) is 1.0 (**a**), 1.5 (**b**), or 2.0 mm (**c**) (width (W), 50 mm; length (L), 100 mm).

**Figure 13:**
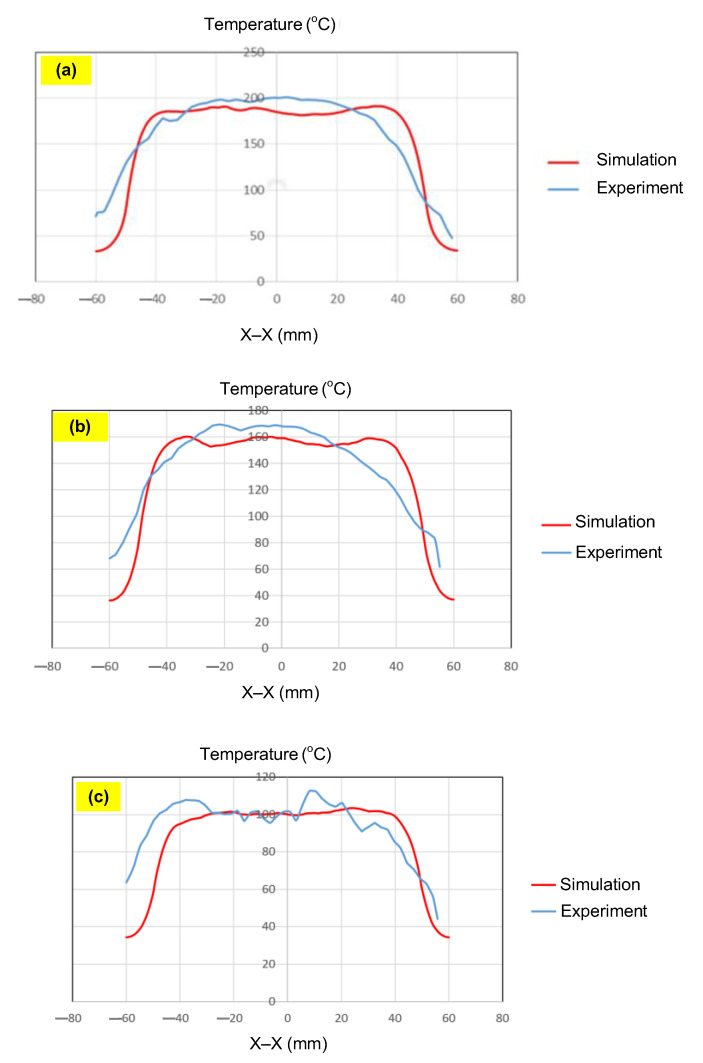
Comparison of temperature on the X–X line between simulated and experimental measurements for the case where the thickness is 1.0 mm (**a**), 1.5 mm (**b**), or 2.0 mm (**c**) (width (W), 50 mm; length (L), 100 mm).

To validate the results obtained with the simulation model, induction heating experiments with a fixed heating time of 5 s were performed on three cavity inserts with different thicknesses (1.0, 1.5, and 2.0 mm), while the width and length were kept constant at 50 and 100 mm, respectively. The comparison of the simulation and experimental results (Figure 12 and Figure 13) demonstrated strong agreement, confirming the feasibility of the proposed approach.

For the thinnest insert (1.0 mm, Figure 13a), the temperature distributions obtained through the simulation and experiment were nearly identical. The maximum temperature reached approximately 200 °C, with a deviation of only about 5 °C. Both approaches showed the highest temperature at the center, decreasing towards the edges, without evidence of edge overheating. However, the experimental profile exhibited smoother transitions at the edges and slightly higher peak values, whereas the simulation predicted a broader plateau region at the center.

When the thickness increased to 1.5 mm (Figure 13b), the maximum temperature dropped to about 160 °C, reflecting the greater thermal mass and energy dispersion. The difference between simulated and experimental values remained below 10 °C, and both confirmed uniform temperature distributions without excessive edge heating. Cross-sectional analysis revealed that the experimental results showed more pronounced variations at the boundaries, while the simulation results continued to underestimate the maximum peak.

For the thickest insert (2.0 mm, Figure 13c), the maximum temperature was only about 100 °C, with the deviation between the two methods remaining within 10 °C. The temperature distribution consistently peaked at the center and decreased towards the edges. The experimental results showed a greater fluctuation in the thermal curve, whereas the simulation results were more stable but tended to predict lower peak values.

Overall, across all three cases, the simulation successfully replicated the key heating trends: central concentration, symmetrical distribution, and gradual decline toward the edges. The main discrepancies were associated with maximum temperature values, which the simulation results typically underestimated, particularly for thicker inserts. These differences can be attributed to the idealized boundary conditions and simplified treatment of convective and radiative heat transfer, as well as the assumption of constant material properties in simulations. Experimental uncertainties in temperature measurement may also have contributed to the observed deviations.

Despite these limitations, the results confirm that the numerical model provides reliable predictions of induction heating behaviors. It not only captures the characteristic thermal concentration at the insert center but also enables quantitative evaluation of geometric parameters such as insert thickness. With further refinement to incorporate more realistic boundary conditions and additional heat transfer mechanisms, the model could achieve a higher accuracy, making it a practical tool for the design and optimization of induction heating systems. Thus, the comparison of the simulation and experimental results demonstrated that numerical modeling can serve as a dependable approach for predicting, controlling, and optimizing induction heating processes, ultimately improving quality and stability in the context of thin-walled injection molding production.

### 3.2. Application of Localized Induction Heating to Enhance Mold Filling in Thin-Walled Injection Molding

Building on the findings presented in Section 3.1, where the localized heating of mold inserts was evaluated using the evaluation model (Figure 2a), this study applied the concept to an application model (Figure 2b) to analyze induction heating inside the mold cavity and its effect on thermal distribution over time.

#### 3.2.1. Internal Induction Heating for Mold Cavity Applications

Numerical simulations were conducted to examine the temporal evolution of temperature fields under internal induction heating. The results (Table 10) revealed that both the peak temperature and the spatial distribution of the thermal field varied significantly as the heating time increased from 1 s to 9 s.

At 1 s, the maximum temperature was about 170 °C, which was concentrated mainly near the surface adjacent to the induction coil, while most of the cavity remained relatively cold. By 2 s, the temperature rose to approximately 262 °C and the heated zone began to expand into the cavity, although a pronounced gradient still existed between the center and the edges. At 3 s, the maximum temperature increased sharply to 340 °C, with the hot zone extending laterally, indicating the stronger contribution of thermal conduction. After 5 s, the peak reached around 430 °C, with the hot region surrounding the central gate hole and stretching toward the cavity edges, reflecting a strong energy concentration effect. By 7 s, the maximum temperature was about 500 °C, and nearly the entire cavity surface was covered by a high-temperature field, although localized hot spots remained at the center and edges. At 9 s, the system approached a quasi-saturated state, with a maximum temperature of 555 °C. The thermal distribution became more symmetrical, and the gradient from center to edges was significantly reduced. However, due to edge effects, the cavity edges and central gate hole remained hotter, caused by the concentration and bending of magnetic flux lines, which increased the induced current density and Joule heating.

The simulations also indicated that the upper edge of the insert consistently heated faster and reached higher temperatures than the lower edge. This asymmetry can be attributed to geometric positioning, as the upper section lies within a stronger magnetic field, generating greater induced current density and, consequently, more heat.

In summary, extending the heating time from 1 s to 9 s increased the maximum temperature from 170 to 555 °C while significantly improving the temperature uniformity. Differences between the cavity center and edges, as well as between upper and lower boundaries, diminished over time due to the balancing effect of thermal conduction. Nevertheless, the persistence of localized hot spots suggests that optimizing the heating time alone is insufficient. A combined approach, including coil configuration adjustments, is essential to achieve both high heating efficiency and uniform temperature distribution, ensuring high product quality and process stability in the context of thin-walled injection molding.

Table 11 illustrates the evolution of temperature distribution within the mold cavity under internal induction heating at different heating durations. The infrared thermographs clearly show that both the maximum temperature and the uniformity of the thermal field depend strongly on the heating time and the subsequent cooling period after power shutoff. As the heating duration increased from 1 s to 3 s, the maximum temperature rose sharply from 140 to 340 °C, indicating the high heating efficiency of the induction system. Correspondingly, the temperature decay during the cooling stage was also faster at higher temperatures, while the overall thermal retention improved with longer heating times.

After 1 s of heating, the temperature field was weak and localized, with a rapid temperature drop to 125 °C after 2 s and 107 °C after 5 s of cooling, revealing limited heat storage within the mold insert. At 2 s of heating, the maximum temperature increased to 239 °C and remained above 195 °C after 5 s, showing improved heat accumulation and slower cooling behavior. The 3 s case achieved the highest surface temperature of 340 °C, decreasing to 315 and 250 °C after 2 and 5 s, respectively, while still maintaining the highest overall thermal energy.

Spatially, the temperature distribution at the end of each heating cycle was non-uniform, with hot zones concentrated near the edges where the induction coil was most effective. During the subsequent 2–5 s, heat diffused inward, resulting in a more uniform temperature field across the cavity surface. This transient behavior demonstrates that heat conduction continues even after the power supply is turned off, supporting improved flowability during the early stages of melt filling.

In summary, Table 11 highlights that the heating duration is a critical process variable controlling both the peak cavity temperature and its temporal decay. Short heating durations produce insufficient and uneven heating, while excessive durations may lead to unnecessary thermal accumulation. Therefore, a heating time of approximately 2–3 s provides an optimal balance between high surface temperature and moderate cooling rate, ensuring both efficiency and stability in the injection molding process.

Table 12 compares the temperature distribution between the simulated and experimental results for different induction heating durations (1, 2, and 3 s). Overall, both approaches exhibited a consistent trend, confirming the reliability of the simulation model in predicting the thermal behavior of the mold insert. At 1 s of heating, the maximum temperatures reached approximately 170 °C in the simulation and 140 °C in the experiment, showing a moderate difference that can be attributed to transient measurement effects and heat losses in the actual setup. When the heating duration increased to 2 s, the temperatures rose to 262 °C (simulation) and 240 °C (experiment) and, at 3 s, the respective peak values reached 355 and 340 °C. The temperature fields show that heat was concentrated around the central insert area and gradually diffused outward with time.

The slight discrepancies between the simulated and experimental values arose due to several practical factors. First, there is an inevitable delay in temperature acquisition during the experiment due to sensor response time and surface emissivity variations in the infrared camera, which may cause the recorded temperature to lag the actual thermal state. Second, the simulation employed simplified boundary conditions, neglecting factors such as natural convection, radiation losses, and imperfect thermal contact between components, which are difficult to model precisely. Third, the nonlinear temperature-dependent properties of the insert material (e.g., electrical resistivity, magnetic permeability, and specific heat) can alter the actual heat generation rate compared with the constant values assumed in the numerical model. Despite these differences, the overall temperature profiles and peak values between the simulation and experiment were in close agreement, indicating that the coupled electromagnetic–thermal simulation provides an accurate and physically meaningful prediction of the induction heating process.

Table 13 summarizes the predicted surface temperatures of the mold cavity at different heating durations ranging from 0 to 9 s. The temperature increased nearly linearly with time, from 37 °C (no heating) to 180, 220, 260, and 300 °C at 3, 5, 7, and 9 s, respectively. These results highlight the high responsiveness and efficiency of induction heating in achieving a rapid surface temperature increase within a short duration.

Overall, the two tables collectively confirm that the developed induction heating system provides accurate controllability and a fast thermal response, with the simulation data closely matching experimental outcomes. The heating time directly governs the temperature level and the uniformity of its distribution, offering essential guidance for selecting optimal heating durations in thin-walled injection molding applications.

#### 3.2.2. Improving Filling Ability in Thin-Walled Injection Molding

The simulation results (Table 14) revealed that the melt flow length of polypropylene (PP) in thin-walled cavities is strongly influenced by both mold temperature (30–300 °C) and part thickness (0.2, 0.4, 0.6 mm). The general trend indicates that increasing the mold temperature significantly extends the melt flow length, as higher thermal energy reduces viscosity and delays solidification.

For the 0.2 mm specimen, the melt flow length increased from 30.16 mm at 30 °C to 95.79 mm at 300 °C, corresponding to a 217.61% improvement. Nevertheless, even at the highest temperature, complete filling (100 mm) was not achieved, underscoring the inherent challenges of ultra-thin walls with rapid heat loss. The 0.4 mm specimen performed more favorably, rising from 33.77 mm at 30 °C to full filling at 250 °C, representing a 196.14% increase. The 0.6 mm specimen also reached full filling at 250 °C, increasing from 27.35 mm at 30 °C to 100 mm, or 265.63%. However, at low-to-intermediate temperatures (≤210 °C), the 0.6 mm specimen exhibited shorter flow lengths than the 0.4 mm specimen and, at certain points, even the 0.2 mm specimen. This suggests that larger volume parts require higher injection pressure, and that localized premature solidification may occur in thicker sections due to heat transfer limitations.

Comparative analysis revealed that the 0.2 mm specimen consistently underperformed, never reaching full filling. The 0.4 mm specimen demonstrated the best overall performance, achieving full cavity filling at 250 °C and maintaining efficiency across a broad temperature range. The 0.6 mm specimen matched the filling performance of the 0.4 mm specimen at higher temperatures but lagged at lower ones, reflecting less favorable thermal–flow balance. Thus, a thickness of 0.4 mm appears to be the most practical choice, effectively leveraging the benefits of mold heating while avoiding severe heat losses or excessive resistance to flow.

These findings are consistent with previous studies. Chen et al. [11] observed that increasing the cavity gap from 1 to 3 mm reduced the temperature from 166.7 to 155.4 °C, illustrating challenges in thermal control across thickness variations. Muszyński et al. [3] reported standard deviations in mold temperature of up to 34.3 °C under high current conditions, particularly near the edges, which affected filling uniformity. With PP, raising the mold temperature from 30 to 110 °C improved the melt flow length by 25.5% for 1 mm parts [30]. Uyen et al. [31] demonstrated that localized heating at the gate from 200 to 400 °C enhanced filling of 0.2 mm parts from 65.4% to 100%. Similarly, Minh and Le [32] found that the ABS melt flow length increased from 71.5 to 168.1 mm when the induction heating time was increased from 0 to 5 s.

A summary of the effects of mold temperature on filling ability across different thicknesses is presented in Table 14. Overall, mold temperature is the dominant factor in determining the flow length, while wall thickness dictates efficiency. The 0.4 mm specimen emerged as the optimal design choice, ensuring full filling at 250 °C and consistent performance across a wide temperature range. In contrast, 0.2 mm parts require supplementary measures for full filling, such as increased injection pressure, higher melt temperature, or advanced thermal control techniques. Meanwhile, thicker (0.6 mm) parts can achieve complete filling under sufficient heating but remain less effective at lower temperatures. Collectively, these results provide strong quantitative evidence for the interplay of mold temperature and wall thickness, highlighting 0.4 mm as the most practical thickness for achieving optimal filling and obtaining high-quality PP thin-walled products.

#### 3.2.3. Experimental Validation of Filling Ability with Internal Induction Heating

**Figure 14:**
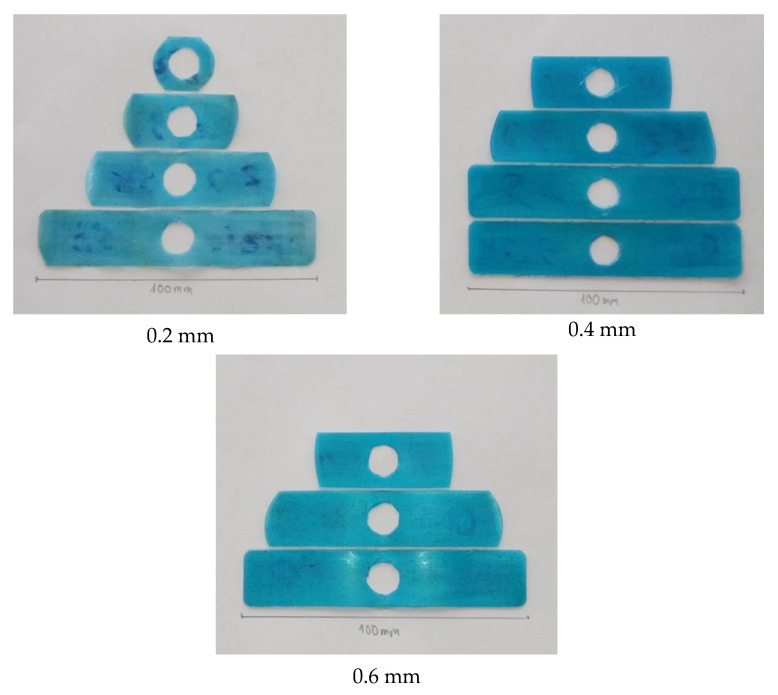
PP plastics with thicknesses of 0.2, 0.4, and 0.6 mm produced under experimental induction heating durations of 0, 3, 5, and 7 s.

Experimental injection molding tests (Figure 14, Table 15) demonstrated that the melt flow length of polypropylene (PP) is strongly dependent on both part thickness (0.2, 0.4, and 0.6 mm) and induction heating duration (0, 3, 5, and 7 s). The general trend shows that longer heating times significantly enhance filling performance by lowering viscosity and delaying polymer solidification.

Table 15 presents the experimental melt flow length results for polypropylene (PP) specimens with three different thicknesses (0.2, 0.4, and 0.6 mm) under various induction heating durations (0, 3, 5, and 7 s). Each condition was measured three times, and the average values are reported. The results clearly demonstrate the influence of heating duration and specimen thickness on flow behavior during injection molding.

Without heating, the melt flow was severely restricted due to rapid cooling at the mold wall, yielding flow lengths of only 22.33, 45.66, and 50.33 mm for the 0.2, 0.4, and 0.6 mm specimens, respectively. When heating was applied for 3 s, the flow lengths increased significantly to 43.66, 83, and 85.33 mm, indicating that even a short heating duration can effectively raise the mold surface temperature and enhance melt fluidity.

At 5 s of heating, the 0.4 mm specimen achieved nearly complete filling (98.67 mm), while the 0.6 mm specimen reached full filling (100 mm). The 0.2 mm specimen also showed a substantial improvement to 71.66 mm. After 7 s, all specimens achieved full cavity filling (100 mm), confirming that a sufficient heating duration ensures complete mold filling regardless of thickness.

Comparison of the specimens revealed distinct thickness-dependent behaviors. The 0.2 mm specimen consistently showed the shortest flow lengths and required the longest heating duration (7 s) to reach complete filling due to its high surface-to-volume ratio, which accelerates heat loss and premature solidification. The 0.4 mm specimen exhibited the most efficient performance, achieving full filling after only 5 s due to a balanced combination of heat retention and flowability. Although the 0.6 mm specimen retained heat more effectively, it required a longer heating time to achieve a uniform temperature distribution across its thickness.

Overall, the results confirm that induction heating significantly improves melt flow length in the thin-wall injection molding of PP. The optimal condition was found at a heating duration of 5 s for the 0.4 mm specimen, which achieved complete filling with minimal energy input and process time.

These findings align with previous studies, where mismatched temperatures between molten polymer and mold surfaces were identified as a primary cause of short-shot defects [3]. Other reports also confirmed that extending the induction heating duration from 3 to 7 s substantially increases flow length, particularly in ultra-thin parts. Moreover, optimized heating conditions have been shown to improve the fiber orientation and length, thereby enhancing mechanical properties [33,34,35].

In summary, the induction heating duration exhibited a direct and positive influence on the melt flow length of PP, following a nearly linear trend with time. Among the three thicknesses tested, the 0.4 mm specimen demonstrated the most efficient flow behavior, achieving complete cavity filling after only 5 s of heating. This superior performance arises from a balanced interaction between thermal retention and flow resistance.

From a thermal perspective, the 0.4 mm wall provided sufficient heat storage to maintain the melt temperature above its solidification threshold during flow, preventing the premature freezing that typically occurs in thinner walls (0.2 mm). Conversely, the thicker wall (0.6 mm) possessed greater thermal inertia, which slowed down the heating rate and caused temperature non-uniformity across its thickness.

From a rheological standpoint, the 0.4 mm thickness offered an optimal flow channel size that minimized shear stress and maintained a stable pressure gradient, whereas the 0.2 mm sample experienced excessive viscous resistance and the 0.6 mm sample suffered from slower heat diffusion that delayed full flow development.

Therefore, the 0.4 mm insert achieved the most favorable combination of heat retention, viscosity control, and flow stability, confirming that an appropriate wall thickness, in conjunction with optimized heating duration, is essential to improving both the mold filling efficiency and product quality in the context of thin-walled polymer injection molding.

#### 3.2.4. Comparison of Simulation and Experimental Filling Performance

**Figure 15:**
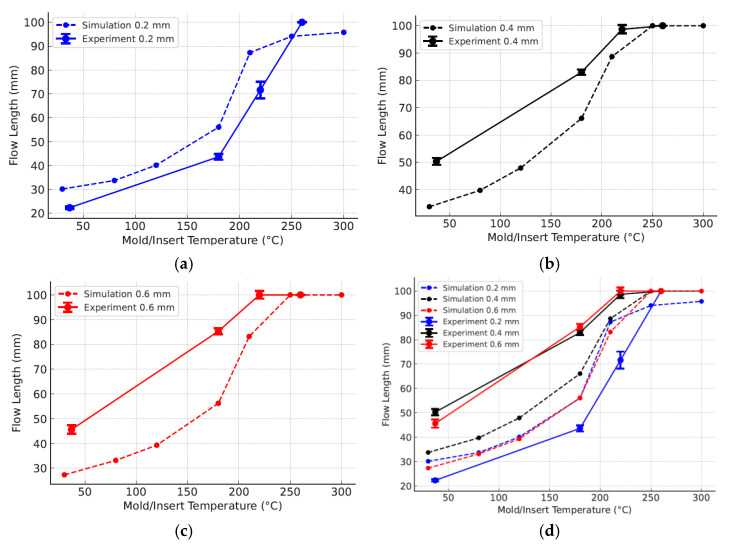
Comparison of simulation and experimental results for cavity filling of PP parts with part thicknesses of 0.2 mm (**a**), 0.4 mm (**b**), and 0.6 mm (**c**) individually, as well as all three together (**d**).

A comparison of the Moldex3D simulation and experimental injection molding results (Figure 15, Table 16) demonstrated the strong effects of mold temperature and part thickness on polypropylene (PP) flow length. For all wall thicknesses (0.2, 0.4, 0.6 mm), longer induction heating raised the mold temperature, reduced viscosity, and delayed solidification, thereby extending flow length.

At 0 s of heating, the flow lengths were restricted: 22.33 mm for 0.2 mm (Figure 15a), 50.33 mm for 0.4 mm (Figure 15b), and 45.66 mm for 0.6 mm (Figure 15c). With 3 s of heating, the values increased sharply to 43.66, 83, and 85.33 mm, respectively. At 5 s, the 0.4 and 0.6 mm specimens nearly achieved full filling (98.67 and 100 mm), while the 0.2 mm specimen remained at 71.66 mm. Complete filling was obtained for all thicknesses by 7 s. These results confirm the strong role of heating time and mold temperature in improving filling performance.

Comparison with the Moldex3D results revealed deviations mainly at low and medium mold temperatures (Figure 15d). For thicker parts, the experiments consistently produced longer flow lengths than the simulations. At 180 °C equivalent, the 0.4 mm specimen reached 83 mm experimentally versus 66.12 mm in the simulation, while the 0.6 mm specimen achieved 85.33 mm compared with 56.23 mm. This difference reflects the limitation of material data in Moldex3D, which is usually obtained at mold temperatures below 100 °C, leading to underestimations of flowability at higher temperatures. In contrast, for the 0.2 mm specimen, the experiments yielded shorter flow lengths than the simulations (43.66 vs. 56.09 mm at 180 °C) due to rapid heat loss and early solidification not being fully captured in the latter.

## 4. Conclusions

This study proposed Internal Induction Heating (In-IH) as a localized mold temperature control technique and investigated its ability to improve the quality and efficiency of thin-walled polypropylene (PP) injection molding processes. Unlike conventional heating methods, which are slow, energy-intensive, and lack local precision, In-IH integrates the insert directly into the induction circuit, enabling rapid and targeted heating of the cavity surface.

Numerical simulations using the COMSOL Multiphysics software enabled an assessment of the effects of insert geometry and boundary conditions, with the results confirming that thinner inserts achieved much higher peak temperatures (~550 °C at 0.5 mm vs. ~80 °C at 2.0 mm), while narrow inserts concentrated heat more effectively (>300 °C at 25 mm vs. ~100 °C at 100 mm). Meanwhile, longer inserts improved uniformity, but reduced peak temperatures by ~20–25%. Increasing the coolant temperature from 10 to 70 °C reduced the center–edge thermal gradient from 60–70 to 15–20 °C. Experimental tests validated these trends, with small deviations due to real heat losses.

Molding trials revealed that mold temperature strongly affects filling performance. At 180 °C, the 0.4 mm specimen achieved a melt flow length of 85.33 mm, outperforming both the 0.6 mm (83 mm) and 0.2 mm (43.66 mm) specimens. Overall, the excellent capability of In-IH to promote rapid, localized heating was demonstrated, significantly enhancing filling, surface quality, and production efficiency in the context of thin-walled injection molding.

Despite its advantages, the internal induction heating system also presents several limitations. Integrating the induction circuit within the mold makes the mold structure more complex, requiring additional space for pipes, connectors, and insulation layers. This complexity increases manufacturing costs and the difficulty of maintenance. Moreover, cooling the insert after heating becomes challenging, as the embedded design limits the use of conventional cooling channels, potentially leading to uneven temperature reduction. Another limitation lies in accurately calculating and controlling the heating source, as electromagnetic and thermal interactions depend on multiple factors such as the coil geometry, material properties, and frequency, making precise prediction and optimization of the heat distribution more difficult.

## Figures and Tables

**Table 1:** Simulation parameters for induction heating method.

Physical Property	Density	Electrical Resistivity	Relative Permeability	Specific	Thermal Conductivity
Unit	Kg/m^3^	Ωm	µ	J/kg·K	W/m·K
Air at 25 °C	1.18	-	1	1000	0.0256
Steel	7800	5.5 × 10^−7^	100	420	20
Copper	8940	1.71 × 10^−7^	0.99	392	400

**Table 2:** Simulation parameters for evaluation model.

Physical Property	Electrical Conductivity	Boundary Feed 1	Frequency	Simulation Time
Unit	S/m	A	Hz	s
Value	1 × 10^7^	20	20,000	(0, 0.5, 5)

**Table 3:** Simulation parameters for real cavity model.

Parameter Simulation	
Assigned material	Copper, steel
Setting coil intensity: I	A
Initial texture temperature	30 °C
Type of thermal analysis	Heat transfer in solids
Time-series analysis	0 s to 9 s (step 0.5 s)
Minimum step for analytical time scale	0.01 s
Initial time	0 s

**Table 4:** Experimental parameters.

STT	Insert Thickness t (mm)	Insert Width W (mm)	Insert Length L (mm)	Coolant Temperature (°C)
1	0.5	50	100	30
2	1.0			
3	1.5			
4	2.0			
5	1.0	25		
6		50		
7		75		
8		100		
9		50	50	
10			100	
11			150	
12			200	
13	1.0	50	100	10
14				20
15				30
16				50
17				70

**Table 5:** Properties of induction heating machine.

Parameter	Value
Controller	Programmable Logic Controller (PLC)
Max. power	30 (kW)
Voltage	380 (V)
Current	150 (A)
Cooling method	Water
Thermal sensor	Thermal couple (K-type)

**Table 6:** Statistical heating performance results when investigating the influence of thickness (t).

Thickness (t)	Max. Temperature
0.5 mm	≈550 °C
1.0 mm	≈200 °C
1.5 mm	≈160 °C
2.0 mm	≈100 °C

**Table 7:** Statistical heating performance results when investigating the influence of width (W).

Width (W)	Max. Temperature
25 mm	≈300 °C
50 mm	≈200 °C
75 mm	≈120 °C
100 mm	≈100 °C

**Table 8:** Statistical heating performance results when investigating the influence of length (L).

Length (L)	Max. Temperature
50 mm	≈220 °C
100 mm	≈180 °C
150 mm	≈170 °C
200 mm	≈160 °C

**Table 9:** Statistical induction heating results when investigating the influence of coolant temperature.

Coolant Temperature	T _Heating Temperature_ (Line X–X)
10 °C	≈40 °C
20 °C	≈50 °C
30 °C	≈60 °C
50 °C	≈80 °C
70 °C	≈100 °C

**Table 10:** Simulation results for temperature distribution within the mold cavity.

Heating Time	Temperature Distribution (°C)
1 s	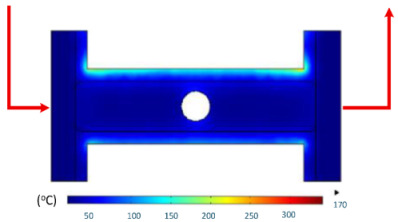
2 s	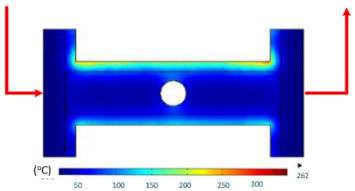
3 s	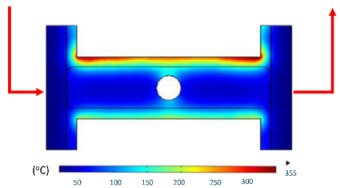
5 s	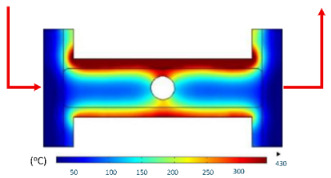
7 s	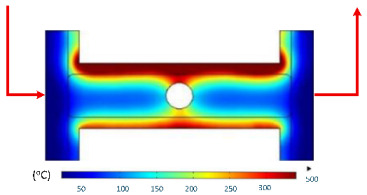
9 s	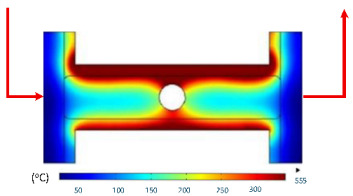

**Table 11:** Temperature distribution within the mold cavity under internal induction heating.

Induction Heating Time	End of Heating Step	2 s After End of Heating Step	5 s After End of Heating Step
1 s	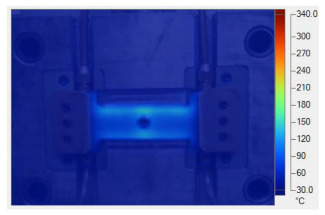 $t_{max}^{{}^{\circ}}$ = 140 °C	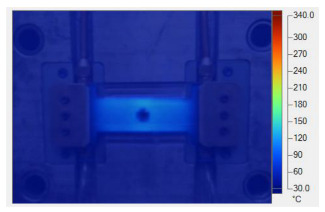 $t_{max}^{{}^{\circ}}$ = 125 °C	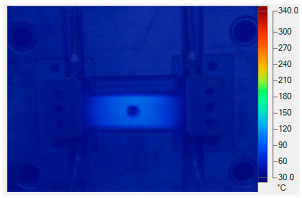 $t_{max}^{{}^{\circ}}$ = 107 °C
2 s	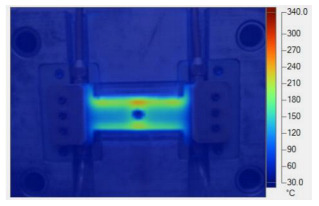 $t_{max}^{{}^{\circ}}$ = 239 °C	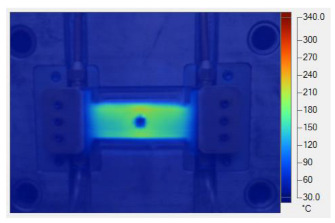 $t_{max}^{{}^{\circ}}$ = 213 °C	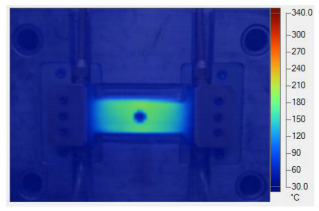 $t_{max}^{{}^{\circ}}$ = 195 °C
3 s	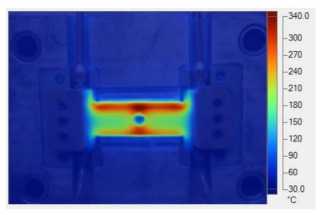 $t_{max}^{{}^{\circ}}$ = 340 °C	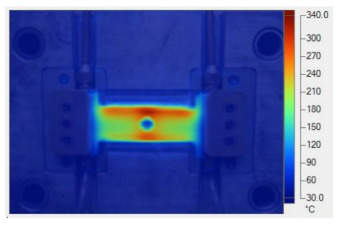 $t_{max}^{{}^{\circ}}$ = 315 °C	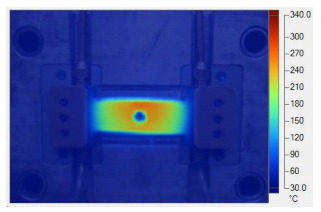 $t_{max}^{{}^{\circ}}$ = 250 °C

**Table 12:** Comparison of temperature distributions from simulation and experimental results.

Heating Time	Simulation	Experiment
1 s	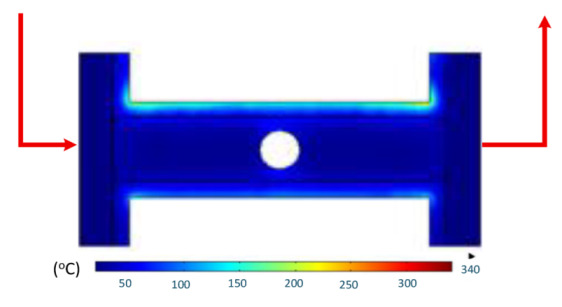 Max: 170 °C	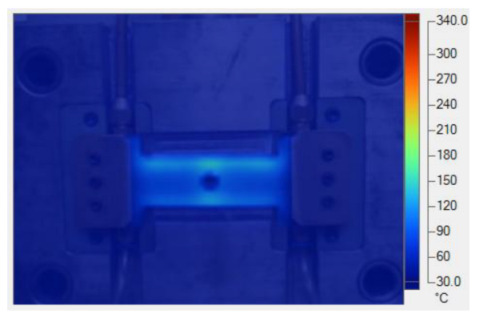 Max: 140 °C
2 s	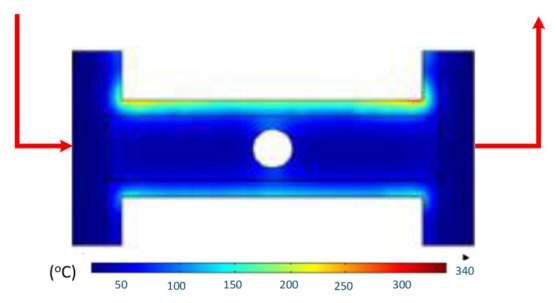 Max: 262 °C	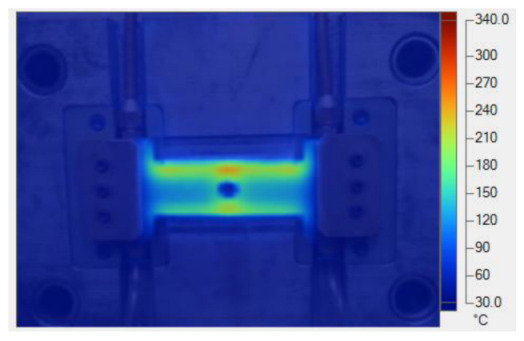 Max: 240 °C
3 s	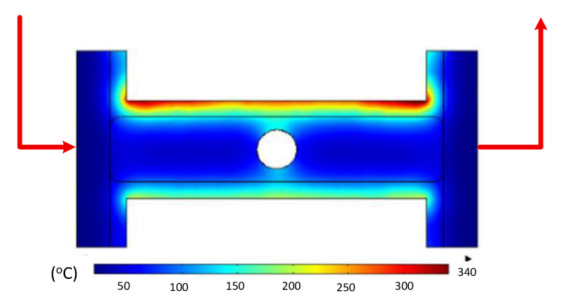 Max: 355 °C	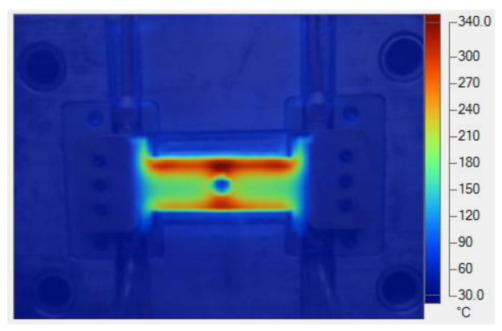 Max: 340 °C

**Table 13:** Mold cavity temperatures achieved at different induction heating durations.

Heating Time	0 s	3 s	5 s	7 s	9 s
Insert Surface Temperature	37 °C	180 °C	220 °C	260 °C	300 °C

**Table 14:** Simulation results of the melt flow length for PP plastics.

Old Cavity Temperature	0.2 mm Mold Cavity	0.4 mm Mold Cavity	0.6 mm Mold Cavity
30 °C	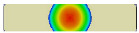 L = 30.16 mm	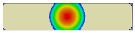 L = 33.77 mm	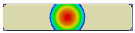 L = 27.35 mm
80 °C	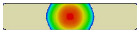 L = 33.73 mm	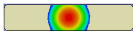 L = 39.77 mm	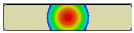 L = 33.17 mm
120 °C	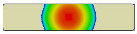 L = 40.14 mm	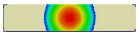 L = 47.94 mm	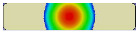 L = 39.33 mm
180 °C	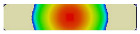 L = 56.09 mm	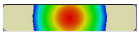 L = 66.12 mm	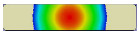 L = 56.23 mm
210 °C	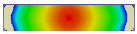 L = 87.29 mm	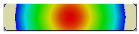 L = 88.69 mm	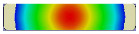 L = 83.21 mm
250 °C	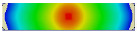 L = 94.11 mm	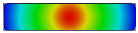 L = 100 mm	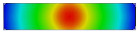 L = 100 mm
300 °C	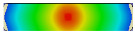 L = 95.79 mm	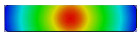 L = 100 mm	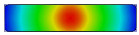 L = 100 mm

**Table 15:** Experimental results for melt flow length in the mold cavity for PP plastics.

		Length (mm) (Experiment 1)	Length (mm) (Experiment 2)	Length (mm) (Experiment 3)	Length (mm) (Average Value)
Ex.PP_0.2 mm	0 s	22	23	22	**22.33**
	3 s	45	42	44	**43.66**
	5 s	71	68	76	**71.66**
	7 s	100	100	100	**100**
Ex.PP_0.4 mm	0 s	52	49	50	**50.33**
	3 s	84	82	83	**83**
	5 s	99	100	97	**98.67**
	7 s	100	100	100	**100**
Ex.PP_0.6 mm	0 s	44	45	48	**45.66**
	3 s	87	84	85	**85.33**
	5 s	100	100	100	**100**
	7 s	-	-	-	**-**

**Table 16:** Comparison of simulated and experimental melt flow length results.

	Stamp Temperature						
	30 °C	80 °C	120 °C	180 °C	210 °C	250 °C	300 °C
**Si.PP_0.2 mm**	30.16 mm	33.73 mm	40.14 mm	56.09 mm	87.29 mm	94.11 mm	95.79 mm
**Si.PP_0.4 mm**	33.77 mm	39.77 mm	47.94 mm	66.12 mm	88.69 mm	100 mm	100 mm
**Si.PP_0.6 mm**	27.35 mm	33.17 mm	39.33 mm	56.23 mm	83.21 mm	100 mm	100 mm
	**Insert Temperature (°C)**						
	37 °C	180 °C	220 °C	260 °C			
**Ex.PP_0.2 mm**	22.33 mm	43.66 mm	71.66 mm	100 mm			
**Ex.PP_0.4 mm**	50.33 mm	83.0 mm	98.67 mm	100 mm			
**Ex.PP_0.6 mm**	45.66 mm	85.33 mm	100 mm	100 mm			

## Data Availability

The data used to support the findings of this study are available from the corresponding author upon request.
